# Quantification of topological features in cell meshes to explore E-cadherin dysfunction

**DOI:** 10.1038/srep25101

**Published:** 2016-05-06

**Authors:** Tânia Mestre, Joana Figueiredo, Ana Sofia Ribeiro, Joana Paredes, Raquel Seruca, João Miguel Sanches

**Affiliations:** 1 Institute for Systems and Robotics, Instituto Superior Técnico, Lisboa, Portugal; 2 Instituto de Investigação e Inovação em Saúde (i3S), Porto, Portugal; 3 Institute of Molecular Pathology and Immunology of the University of Porto (IPATIMUP), Porto, Portugal; 4 Department of Pathology and Oncology, Medical Faculty of the University of Porto, Porto, Portugal

## Abstract

In cancer, defective E-cadherin leads to cell detachment, migration and metastization. Further, alterations mediated by E-cadherin dysfunction affect cell topology and tissue organization. Herein, we propose a novel quantitative approach, based on microscopy images, to analyse abnormal cellular distribution patterns. We generated undirected graphs composed by sets of triangles which accurately reproduce cell positioning and structural organization within each image. Network analysis was developed by exploring triangle geometric features, namely area, edges length and formed angles, as well as their variance, when compared with the respective equilateral triangles. We generated synthetic networks, mimicking the diversity of cell-cell interaction patterns, and evaluated the applicability of the selected metrics to study topological features. Cells expressing wild-type E-cadherin and cancer-related mutants were used to validate our strategy. Specifically, A634V, R749W and P799R cancer-causing mutants present more disorganized spatial distribution when compared with wild-type cells. Moreover, P799R exhibited higher length and angle distortions and abnormal cytoskeletal organization, suggesting the formation of very dynamic and plastic cellular interactions. Hence, topological analysis of cell network diagrams is an effective tool to quantify changes in cell-cell interactions and, importantly, it can be applied to a myriad of processes, namely tissue morphogenesis and cancer.

Cadherins are a superfamily of transmembrane proteins that comprises more than one hundred members in humans, including classical proteins, protocadherins and cadherin-related proteins^1^. The main function of cadherin receptors lies in their contribution to the preserve cell-cell cohesion in solid tissues of the body^1^,^2^. Cell-cell adhesion is crucial for the assembly of individual cells and, therefore, responsible for the formation and maintenance of the normal epithelia architecture^3^.

E-cadherin is the main component of the Adherens Junctions and, as such, the major contributor to adhesion mechanisms^4^,^5^. This transmembrane glycoprotein belongs to the subfamily of classical, also known as type I cadherins, and it is composed by two main structural domains: the extracellular and the cytoplasmic domain^4^,^5^. The extracellular domain establishes a homophilic binding to other E-cadherin molecules on neighbouring cells, while the cytoplasmic domain assembles with catenins, linking this protein complex to the actin cytosqueleton and, consequently, ensuring a regular epithelia structure^4^,^5^,^6^,^7^,^8^. The combination of mechanical and signal-transducer properties of E-cadherin is responsible for cell-cell aggregation and suppression of cell invasion, in a process dependent on the presence of Ca^2+^ 9,10. Thus, given the pivotal role of E-cadherin for epithelia homeostasis, it is not surprising that alterations in E-cadherin expression or structural modifications in its encoding gene (CDH1) can result in loss of cell-cell adhesion and in profound epithelia changes that can culminate in highly invasive and lethal diseases such as cancer^2^,^5^,^11^,^12^.

In recent years, a number of quantitative methods to evaluate cellular adhesion abnormalities have been established. Methods such as atomic force microscopy (AFM), fluorescence resonance energy transfer (FRET), fluorescence recovery after photobleaching (FRAP), as well as cell aggregation assays have been used to measure the strength of cadherin-dependent adhesion and detect possible disturbances of cell-cell attachment mechanisms^13^,^14^,^15^,^16^,^17^,^18^. However, aside of being difficult to implement and unable to quantify cell-cell adhesion within their natural context, these methods also fail in determine the consequences of loss of adhesion within a tissue. Therefore, the development of alternative methods addressing this issue became an urgent need in cell biology field.

In this work, we propose a quantitative imaging tool to detect abnormal epithelial organization, based on 2D *in situ* microscopy images of cells stained with DAPI. We used cell nucleus staining to create artificial cellular networks, from which we could extract quantitative data regarding cell distribution patterns, intercellular distance and cell-cell contact distortion. To validate the accuracy of our strategy, cells expressing wild-type (WT) E-cadherin and a panel of cancer-related E-cadherin mutants, leading to aberrant E-cadherin expression and impacting adhesion competence were used^16^,^19^,^20^,^21^,^22^,^23^,^24^,^25^.

## Results

### Network design

In this work, we developed a quantitative method to evaluate morphological and structural effects of adhesion loss. For that purpose, cell-based graphs (networks) were created using images of DAPI-stained cells and connecting triplets of neighbouring cells. An efficient analytical pipeline for the network was then developed and validated in a well-known model of loss of cell adhesiveness^15^,^16^,^20^,^21^,^22^,^23^,^26^,^27^,^28^,^29^,^30^,^31^,^32^.

**Figure 1:**
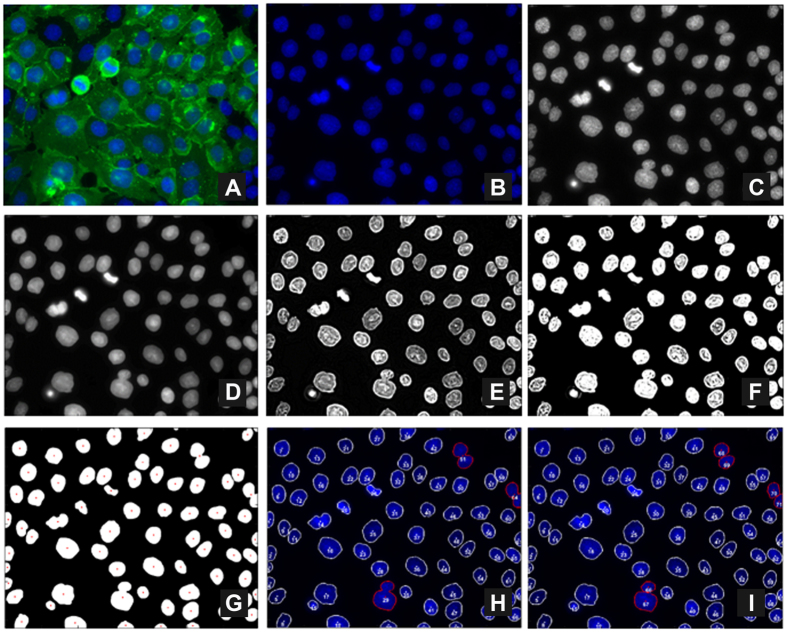
Image processing and nuclei segmentation pipelines. (**A**) RGB original fluorescence image; (**B**) Blue channel of the image; (**C**) Gray-scale version of image (**B**); (**D**) Denoised image; (**E**) Contrast increase of image (**D**); (**F**) Intensity adjustment; (**G**) Otsu method output from image (**F**); (**H**) Nuclei segmentation output; (**I**) Manual fixation of nuclei that were not properly segmented (nuclei with red contour).

**Figure 2:**
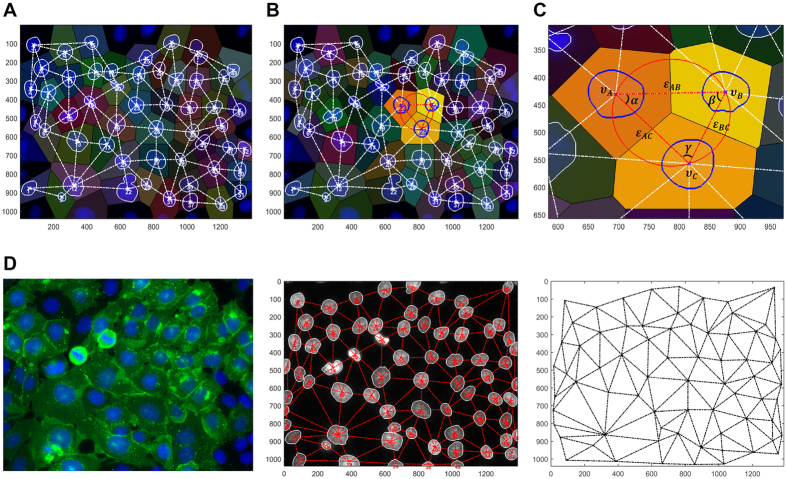
Cell network design. (**A**) Delaunay Triangulation superimposed with Voronoi diagram based on nuclei geometric centres. (**B**) Cellular networks are composed by clusters of three cells, forming triangles. (**C**) Triangle geometric features such as centroides (***v***_*A*_, ***v***_*B*_, ***v***_*C*_), edges (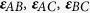) and angles (*α*, *β*, *γ*) that were used to develop a network analytical pipeline. (**D**) On the left, cells expressing WT human E-cadherin immunostained with anti-human E-cadherin antibody (green) and nuclei counterstained with DAPI (blue). Cell nuclei overlapped with the corresponding intercellular network, at the centre. The resulting network is showed on the right.

As a first approach, denoising and nuclei segmentation was performed in each image by application of the Otsu method and the Moore-Neighbor tracing algorithm, modified by Jacob’s stopping criteria (Fig. 1, details in Materials and Methods section). Subsequently, nuclei geometric centre (***υ***) was computed and its definition allowed the establishment of a segments connecting two neighbouring nuclei (***ε***) and, thus, the creation of an undirected graph, *G* (***υ, ε***), by sequential association of other neighbours (Fig. 2A,D). In fact, a network could be designed using the Delaunay triangulation algorithm^33^ which produced a cluster of triangles (triangular mesh) with vertices ***υ***_***k***_. Among all possible triangular mesh configurations for a given set of nodes (nuclei centres), this algorithm selects the one that maximizes the smaller angle of the triangles. Hence, this criterion defines the neighbours of a specific point.

Occasionally, highly obtuse triangles, containing large (≫*π*/2) and small (≪ *π*/2) angles, are generated by the Delaunay tessellation and represent outliers. Therefore, we established that those triangles with
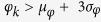
 or 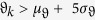 are removed from the mesh and are thus not considered for analysis.

Noteworthy, with our approach, the resulting network do not depend on size or shape of nuclei and cytoplasm, overcoming possible difficulties that arise from segmenting individual cytoplasm*s,* and circumventing additional fluorescence labelling of cells and plasma membranes.

As showed in Fig. 2D, the network obtained is constituted strictly by triangles which accurately represent cell distribution and cell-cell interaction patterns.

### Network quantitative analysis

Taking this into account, we explored triangle geometric features such as vertices, length of the edges, angles and area, to develop a quantitative system for topological analysis of the networks.

As represented in Fig. 2C, our method postulates that each triangle is defined by a triplet of vertices (***v***_*A*_, ***v***_*B*_, ***v***_*C*_), a triplet of edges (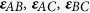) with length 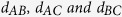, and that three angles are formed between them (*α*, *β*, *γ*).

The length of the edges, ***ε***_*ij*_ =  ***v***_*j*_ *− **v***_*i*_ in which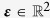, is an Euclidean norm


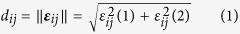


Further, the angle between the edges ***ε***_*i*_ and ***ε***_*j*_ was determined as follows


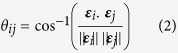


Triangle areas were calculated using the formula


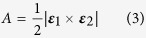


in which ***ε***_1_ ×  ***ε***_2_ is the product of two of the triangle edges.

To assess network regularity, two different metrics – length distortion and angle distortion – were computed and defined as follows


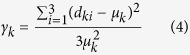



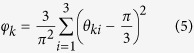


Here, *k* =  1… *N*_*T*_, being *N*_*T*_ the total number of triangles of a mesh, and 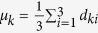, the mean length of the triangle edges.

*γ*_*k*_ measures the length variance of the *k*^*th*^ triangle when compared to the ideal equilateral triangle, for which *γ*_*k*_ =  0. This metric is invariant to the size of triangles due the term 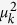 at the denominator. Similarly, *φ*_*k*_ measures the variance of the angles in comparison with an equilateral shape.

Overall, our strategy proposes four parameters to characterize the cell-based networks: i) edges length (*d*_*ij*_); ii) triangle area (*A*_*i*_); iii) length distortion (*γ*_*k*_); and iv) angle distortion (*φ*_*k*_).

### Synthetic networks reflect the diversity of cell spatial distribution

In order to test the sensitivity of our parameters for evaluation of cellular connection patterns, synthetic networks were generated and distorted under controlled conditions.

**Figure 3:**
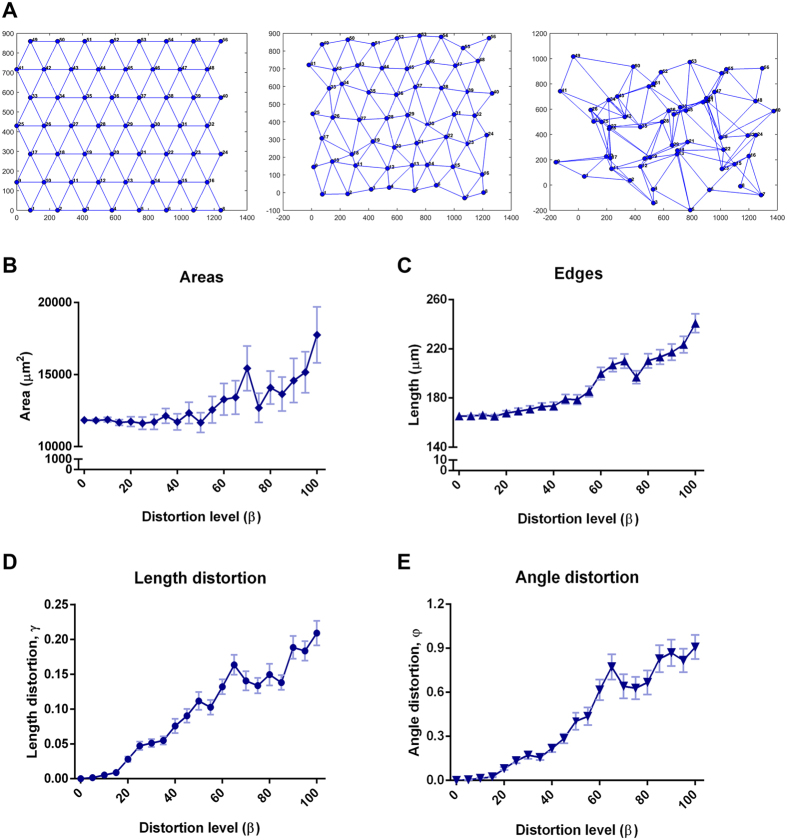
Synthetic networks reflect the diversity of cell distribution patterns. (**A**) Randomly generated synthetic networks mimicking different cellular heterogeneity levels: β  =  0 on the left, β  =  20 in the centre and β  =  100 on the right. Area (**B**), edges length (**C**), length distortion (**D**), and angle distortion (**E**) upon modulation of the network distortion level.

Mimicking the networks derived from the fluorescence microscopy images, these synthetic networks were strictly composed by triangles and the distribution of the nodes was performed based on a pre-defined number of points (#56) in a plane, in such way that the final size of these synthetic planes would correspond to the size of the microscopy images (Fig. 3A). Subsequently, random alterations of the original nodes (***p***_*k*_) were imposed as follows


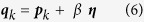


Here, ***η*** ~ *N*(**μ**, *I*) is a bivariate Gaussian distribution with zero mean (μ  =  [0 0]^T^) and a 2 ×  2 identity covariance matrix. β is the parameter used to control the distortion level of the network. A homogeneous network is characterized by β  =  0 and the most heterogeneous network displays β  =  100 (Fig. 3A).

We verified that higher levels of distortion correlate with increased areas and edges length (distances), as well as with higher length and angle distortions, resembling a more heterogeneous and disorganized pattern of cell-cell connections (Fig. 3B–E).

### E-cadherin pathogenic mutations induce higher cellular heterogeneity and abnormal epithelia organization

**Figure 4:**
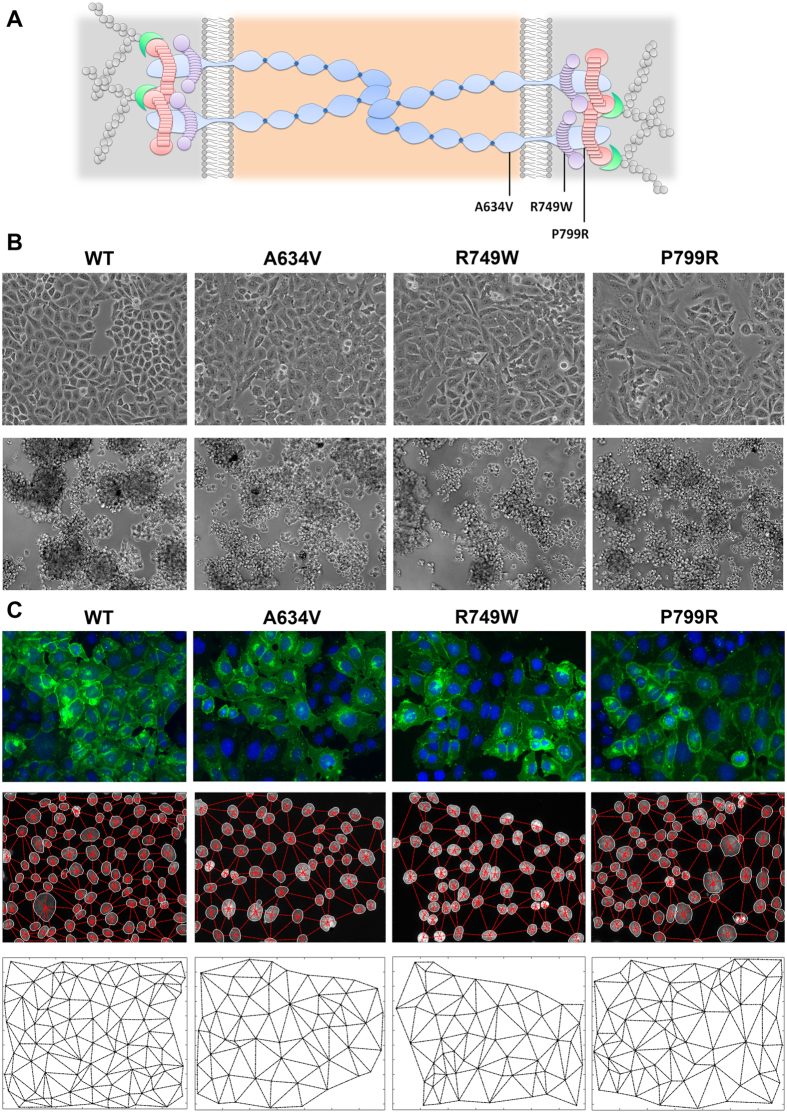
E-cadherin pathogenic mutations generate abnormal patterns of cell distribution. (**A**) Illustration of E-cadherin structure and organization. E-cadherin mature protein comprises a large extracellular domain composed by 5 cadherin repeats, a transmembrane segment and a cytoplasmic domain. The cytoplasmic domain of E-cadherin *bridges* the actin cytoskeleton through a complex of proteins including p120-, β - and α -catenins. The location of the germline E-cadherin mutations used in this study is represented. (**B**) *Phase contrast* microscopy images of CHO cells stably transfected with the WT, A634V, R749W or P799R hE-cadherin. Cell–cell aggregation ability is showed below. The images shown are representative of three independent experiments. (**C**) In the upper panel, immunofluorescence images of WT and A634V, R749W or P799R cells showing aberrant E-cadherin expression of mutants. E-cadherin is labeled in green and nuclei are counterstained with DAPI (blue). In the middle panel, cell nuclei overlapped with the corresponding network. Final networks from WT and mutant E-cadherin cells are presented in the lower panel.

To test whether our method was able to detect abnormal organizational states as a consequence of adhesion deficiency, we applied our strategy to 2D *in situ* images of cells transfected with WT E-cadherin and three loss-of-function mutants (A634V, R749W and P799R; Fig. 4A)^19^,^20^,^21^,^23^.

Despite of an identical epithelia appearance of mutant and WT cell lines under normal culture conditions, immunofluorescence staining and cell-cell aggregation assays demonstrated that all mutants impair normal E-cadherin membranous expression and cell-cell compaction – both remarkable features of cell-cell adhesion dysfunction – corroborating previous results (Fig. 4B and C upper panel)^16^,^20^,^21^,^22^,^25^.

**Figure 5:**
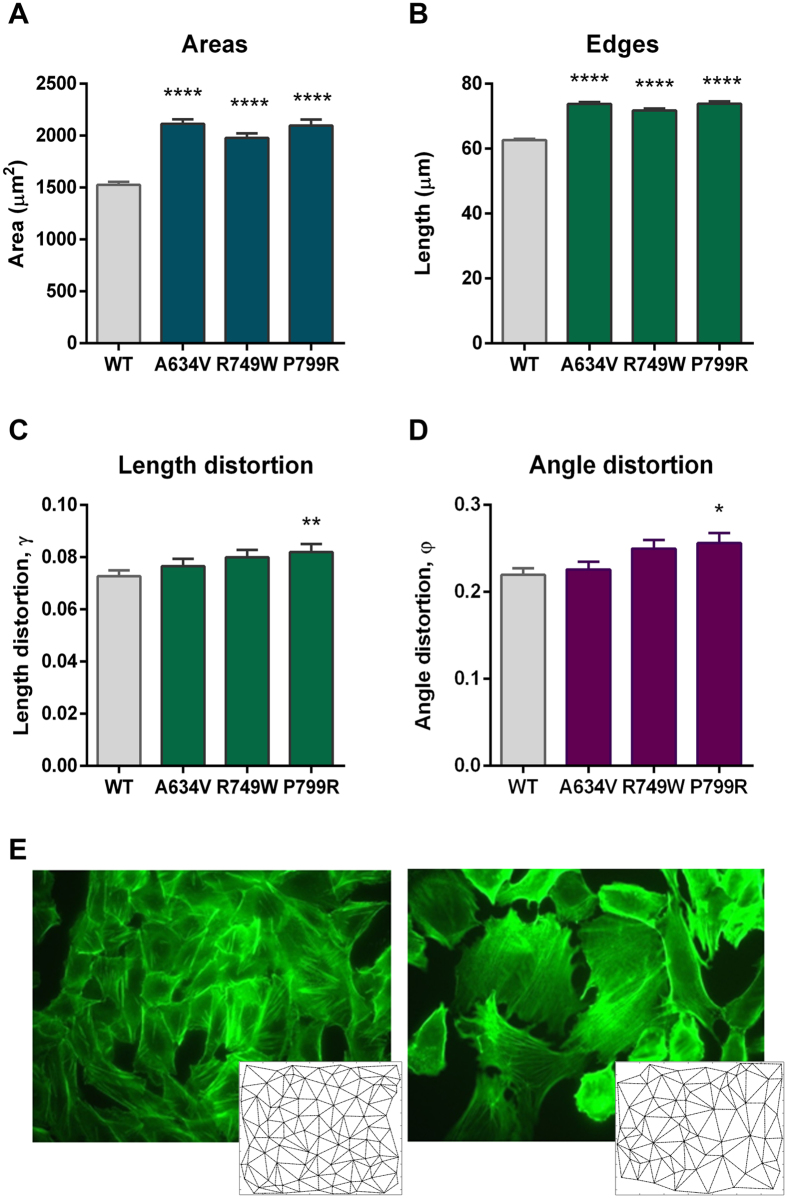
E-cadherin dysfunctional cells display longer and more heterogeneous intercellular connections than the WT E-cadherin cells. Cellular networks obtained from cells expressing WT and mutant E-cadherin were quantitatively analyzed regarding triplet area (**A**), edges length (**B**), length distortion (**C**) and angle distortion (**D**). Graphs show the average ± SE, and *represents p ≤  0.05, **p ≤  0.01, ***p ≤  0.001 and ****p ≤  0.0001. (**E**) FITC-conjugated phalloidin staining of F-actin in WT and P799R expressing cells and the corresponding networks.

As observed in Fig. 4C, the generated networks were able to accurately replicate the distribution pattern of cells. Further, we verified that mutant networks display a more scatter and disorganized phenotype when compared with the regular appearance of the WT network. In fact, network data analysis showed that all E-cadherin mutants present statistically significant higher triplet areas and internuclear distances than the WT E-cadherin-expressing cells (p <  0.0001, Fig. 5A,B). The mean area of the mutants A634V, R749W and P799R was respectively 2115, 1979 and 2098 μ m^2^, while the area of WT cells was 1526 μ m^2^ (Fig. 5A). Moreover, the WT cells are spaced 62.61 μ m in average from each other, whereas the internuclear distance of A634V mutant was 73.77 μ m, R749W was 71.79 μ m and P799R displayed a mean distance of 73.82 μ m. These results indicate that, when compared with WT cells, E-cadherin mutants are loosely attached and present an extended cytoplasm which is suggestive of loss of polarity and increased ability for membrane protrusion formation.

Interestingly, we verified that the P799R mutant, affecting the intracellular portion of E-cadherin close to the β -catenin binding domain, present statistically significant higher length distortion (*γ*_*k*_, p =  0.0069) and angle distortion (*φ*_*k*_, p =  0.016), which are associated to a more irregular networks and disorganized epithelial structures (Fig. 5C,D). By contrast, the extracellular mutant A634V and the juxtamembrane mutant R749W showed less evident length and angle distortions, when compared with the WT counterpart.

These results strongly suggest that E-cadherin mutant P799R present major alterations in cellular organization patterns and we hypothesize that this effect could be due to its inability to bind the actin cytosqueleton. Indeed, by performing the actin staining of P799R and WT cells, we could observe a marked difference in the cytoskeleton structure of both cell types (Fig. 5E). In the presence of the P799R mutation, there is an increased number of stress fibres that span the whole length of the cell, suggesting a stronger and expanded cell-matrix attachment.

Taken together, these findings demonstrate that our analytical strategy applied to cell-based networks is an effective tool to detect and quantify changes in cell-cell interaction and cell distribution patterns that result from E-cadherin loss of function.

## Discussion

Cell-cell adhesion regulates almost all cellular functions by controlling polarity, cell division, intracellular organization and tissue structure^3^,^34^. Loss of adherence is therefore a crucial step for cancer progression by its strong contribution for loss of differentiated characteristics and increased cell invasive capabilities^12^.

**Figure 6:**
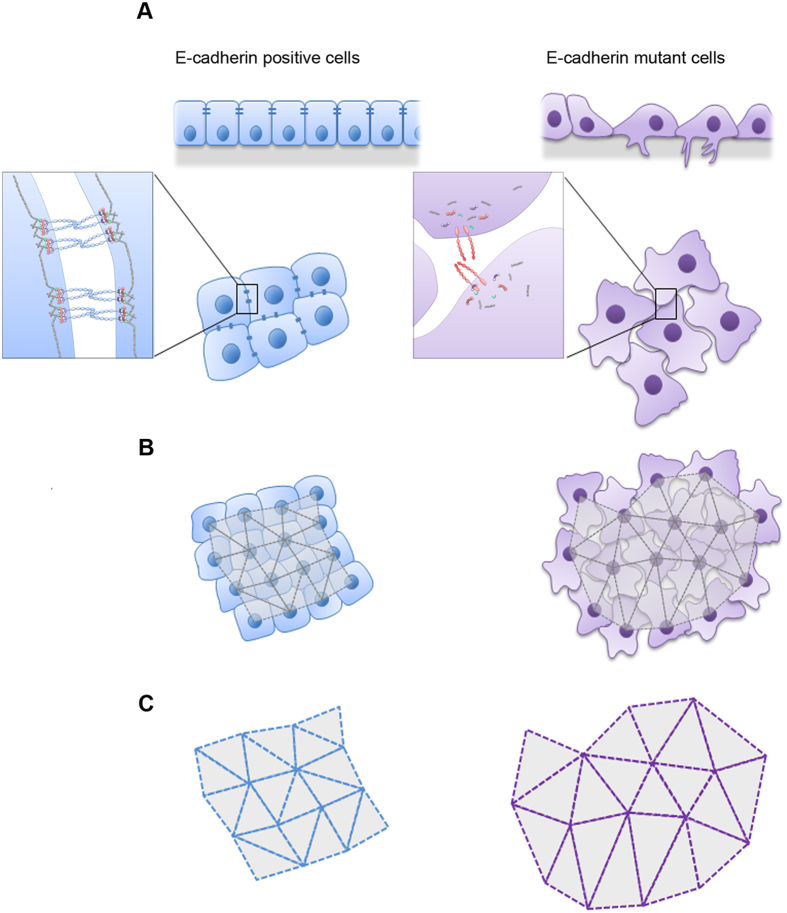
Scheme representing the application of cellular networks to detect cell-cell adhesion dysfunction. (**A**) Cells expressing WT or a pathogenic mutant form of E-cadherin constitute, respectively, well-established models of adhesion competence and impairment. (**B**) Networks from both cell populations can be generated based on *in situ* fluorescence microscopy images of cells with nuclear staining. (**C**) The resulting networks, strictly composed by triangular shapes, are quantitatively analysed and changes in cell-cell connection and cell distribution patterns can be easily identified.

In this work, we propose a quantitative approach, based on 2D *in situ* images, to analyse abnormal cellular distribution patterns that arise as a consequence of loss of cell-cell adhesion mediated by E-cadherin dysfunction. For that purpose, images of cells stained with DAPI were used, enabling the creation of intercellular networks composed by sets of triangles which accurately reproduce cell positioning (geometrical centre of each nuclei) and organization (connection with neighbouring cells/nuclei) in each image (Fig. 6). The processing pipeline applied to each image was composed by a denoising algorithm, followed by contrast and intensity adjustments, nuclei segmentation and geometric cell centres computation, and finally graph construction. An automatically-generated network was thus obtained and further explored.

We established a protocol for quantitative analysis of the network taking advantage of geometric features regarding triangles, namely the area, the length of the edges and the formed angles, as well as their variance, when compared with equilateral triangles (length distortion and angle distortion). The applicability of these parameters was then tested in synthetic networks distorted under controlled conditions, mimicking the diversity of cell-cell interaction patterns and cell spatial distribution of real cell cultures. We found that modelling of distortion parameter allows the generation of more heterogeneous and disordered networks, characterized by higher areas and triangle edges, as well as increased distance and angle variances. These events clearly epitomize episodes of cell-cell adhesion impairment in which cells lose polarity and, consequently, present decreased height, extended cytoplasm and increased cell-matrix interaction (basal cell surface)^35^,^36^,^37^.

A well-known model of adhesion dysfunction, induced by pathogenic E-cadherin mutations associated to hereditary diffuse gastric cancer, was used to validate this hypothesis^16^,^21^,^25^,^28^,^29^,^30^,^32^,^38^,^39^,^40^. Cells expressing WT or different E-cadherin mutants were subjected to our analytical pipeline, and clear differences were found between their distribution patterns. All the mutant networks present a more disorganized spatial distribution when compared with that of the WT cells. In addition, higher triangle areas and edges were produced by the connection of mutant cells.

Interestingly, the P799R mutation, affecting the cytoplasmic domain of E-cadherin, also exhibited higher distance and angle distortions when compared with the WT cell line, suggesting the formation of very dynamic and plastic cellular interactions. In fact, we have previously demonstrated that this mutant affects the binding of E-cadherin to PIPKIγ , a fundamental regulator of E-cadherin function and trafficking, compromising cadherin stability at the plasma membrane^16^,^32^,^41^. An unstable cadherin/catenin complex cannot link the actin cytoskeleton and, as a result, cells lose their polarity, change their epithelial appearance and acquire motile abilities^5^,^9^,^42^. Accordingly, we have showed that P799R cells display an abnormal cytoskeletal organization, suggestive of increased cell-matrix interactions. This cascade effect can even culminate with an abnormal activation of cancer-related signalling such as RTK (receptor tyrosine kinase), WNT and RHO GTPases pathways^5^,^9^,^19^,^32^,^42^.

Despite not being a direct measure of adhesiveness, the study of cellular distribution, through creation of artificial networks, revealed to be very sensitive concerning functional consequences of adhesion loss. Contrarily, assays such as atomic force microscopy (AFM), dual micropipette assay (DPA), fluorescence resonance energy transfer (FRET) and fluorescence recovery after photobleaching (FRAP) provide direct measures of contact strength, however the results do not represent what is observed in real tissues or cell cultures, as these techniques require artificial constructs, specific extracellular matrices or can only be applied in single cell stages^13^.

Different bioimaging tools have been developed to study cell adhesion patterns but so far they were only able to distinguish abrupt phenotype changes. Nawrocki-Raby B. and collaborators proposed an original system to quantify the spatiotemporal collective behavior of different bronchial and mammary epithelial cell lines by using time-lapse videomicroscopy images and graphical geometrical methods^43^. Nonetheless, the authors could only recognize two gross phenotypes among ten different cell lines: a group of highly cohesive cells with a cluster-type spatial distribution, and a second group of very dispersed cells with random spatial distribution^43^. More recently, others have developed a method to characterize cell organization in light microscopy section images of wood^44^. Using a classical Watershed algorithm, it was possible to evaluate cell density and directional arrangements during tissue development and growth, but quantitative data extracted was scarce and poorly explored^44^. In this regard, our method brings new advances to the field since it combines an improved sensitivity to subtle adhesion alterations and an innovative quantitative approach to capture them. In addition, the use of nuclei geometrical centers to generate cell-based graphs is a huge advantage since there is no need for segmenting individual cytoplasms or additional fluorescence labelling of cells and plasma membranes.

Overall, our findings demonstrated that the analysis of cell network diagrams detect and quantify changes in cell-cell connection and cell distribution patterns elicited by E-cadherin dysfunction. Furthermore, this new approach proved to be an easy, fast and reliable technique that can be applied to a wide range of cellular conditions involving cell-cell interactions, maintaining their natural tissue context.

## Materials and Methods

### Cell Culture

Chinese Hamster Ovary (CHO) cells (ATCC number: CCL-61, Barcelona, Spain) stably transfected with vectors encoding the WT or the E-cadherin mutants A634V, R749W and P799R, as previously described^16^, were maintained at 37 °C under 5% CO_2_ humidified air, in α -MEM (+ ) medium (Gibco, Invitrogen) supplemented with 10% fetal bovine serum (HyClone, Perbio), 1% penicillin/streptomycin (Gibco, Invitrogen) and 5 μ g/ml blasticidin (Gibco, Invitrogen).

### Fluorescence Staining

Cells were seeded on 6-well plates on top of glass coverslips and grown to at least 80% confluence. Fixation was performed in ice-cold methanol for 20 minutes, followed by washing and blocking in 5% bovine serum albumin (BSA) in phosphate buffered saline (PBS) for 30 minutes, at room temperature. To determine the pattern of E-cadherin expression in WT and mutant cells, the mouse monoclonal E-cadherin antibody (BD Biosciences) was used at 1:300 dilution in PBS with BSA 5%, and incubated for 1 hour at room temperature. An Alexa Fluor 488 goat anti-mouse (1:500, Invitrogen) was applied for 1 hour in dark, as secondary antibody. The coverslips were mounted on slides using Vectashield (Vector Laboratories) with DAPI. For F-actin staining, cells were fixed in 4% paraformaldehyde (4 °C for 30 minutes) and FITC-conjugated phalloidin (Sigma) was used at 1:500 dilution. Acquisitions were made on a Carl Zeiss Apotome Axiovert 200M Fluorescence Microscope system (Carl Zeiss, Jena, Germany), using × 40 objectives and fixed illumination. Images were captured with an Axiocam HRm camera and the Zeiss Axion Vision 4.8 software.

### Slow aggregation assay

Cell-cell adhesive properties of cells expressing WT and mutant E-cadherin were assessed by slow aggregation assays as described previously^21^. Wells of a 96-well-plate were coated with 50 ml of an agar solution: 100 mg Bacto-Agar (BD Biosciences) in 15 ml of sterile PBS. Upon agar solidification, 200 μ l of a cellular suspension with 1× 10^5 ^cells/ml were seeded in each well (corresponding to 2 ×  10^4^ cells/well). Experimental conditions were always performed in triplicate. The plate was then incubated at 37 °C, in a humidified atmosphere with 5% CO_2_, for 48 h. Aggregation was evaluated under an inverted microscope and photographed with a Nikon (Tokyo, Japan) digital camera.

### Image processing

A pre-processing pipeline was applied to 2D *in situ* images in order to remove noise, increase contrast and adjust the dynamic range of image intensities (Fig. 1 and Supplemental Figure S1).

Denoising was performed as described previously, assuming a Poissonian model for pixel intensity^45^. This algorithm, designed in a Bayesian framework, uses a Log-Total Variation prior favouring piecewise constant solutions for nuclei mask estimation and segmentation purposes. To reduce blur and enhance the contrast between the nuclei and the background, the denoised image was then sharpen by high passing filtering. Finally, image intensity was scaled to reinforce the bi-modality of its histogram and assist the segmentation procedure. A limiting saturation step was applied, in which the highest (*I*_*ij*_ >  0.5) and lowest (*I*_*ij *_<  0.1) intensities were saturated at the maximum (*I*_*ij*_ =  1) and at the minimum (*I*_*ij*_ =  0), respectively. The central range of intensities (0.1 <  *I*_*ij*_ <  0.5), corresponding to transition pixels, was expanded leading to a quasi-binary result. A final binary mask with the non-segmented nuclei was obtained using the classical Otsu method^46^. Small black regions (*I*_*ij*_ =  0) and connected components (white regions) that have fewer than *N* =  60 pixels were removed.

### Nuclei segmentation

The segmentation of each nucleus was performed by computing the contours of the connected components using the Moore-Neighbor tracing algorithm, modified by Jacob’s stopping criteria^46^. Each nucleus contour (*k*), was represented by a sorted list of *n*_*k*_ coordinates, defined as follows


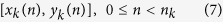


The contour was subsequently filled to produce a binary mask ***b***_*k*_,


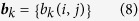


in which


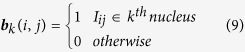


This was obtained using the *poly2mask* function of the image processing toolbox from MatLab. Nuclei not properly segmented were manually fixed. The geometric centres of the segmented nuclei, 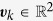, were also computed and calculated from ***b***_*k*_ as follows


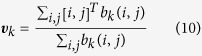


Here [⋅ ]^*T*^denotes the transposition operator.

### Statistical Analysis

Quantitative parameters of WT and E-cadherin mutant cells were statistically analysed using Mann-Whitney tests from *GraphPad Prism* software. In all analysis p <  0.05 was required for statistical significance.

## Additional Information

**How to cite this article**: Mestre, T. *et al.* Quantification of topological features in cell meshes to explore E-cadherin dysfunction. *Sci. Rep.* **6**, 25101; doi: 10.1038/srep25101 (2016).

## Supplementary Material

Supplementary Information
