# Corrigendum: Quantification of topological features in cell meshes to explore E-cadherin dysfunction

**DOI:** 10.1038/srep30222

**Published:** 2016-07-27

**Authors:** Tânia Mestre, Joana Figueiredo, Ana Sofia Ribeiro, Joana Paredes, Raquel Seruca, João Miguel Sanches

Scientific Reports 6: Article number: 25101; 10.1038/srep25101 published online: 05 06 2016; updated: 07 27 2016.

In the original version of this Article, Affiliation 1 was incorrectly given as “Institute for Systems and Robotics, Instituto Superior Técnico, Lisboa, Portugal”. The correct Affiliation is listed below:

“Institute for Systems and Robotics (ISR/IST), LARSyS, Bioengineering Dept, Instituto Superior Técnico, Universidade de Lisboa, Portugal”.

This error has now been corrected in the PDF and HTML versions of this Article.

