# Supplementary material for: Quantification of topological features in cell meshes to explore E-cadherin dysfunction
Source: Sci Rep. 2016 May 6;6:25101. doi: 10.1038/srep25101 (PMC4858654; doi:10.1038/srep25101)
Supplement: Supplementary Information [file srep25101-s1.pdf]

Title: Quantification of topological features in cell meshes to explore E-cadherin dysfunction

Tânia Mestre[1#], Joana Figueiredo[2,3#], Ana Sofia Ribeiro[2,3], Joana Paredes[2,3,4], Raquel Seruca[2,3,4]*, João Miguel Sanches[1]*

[1] Institute for Systems and Robotics, Instituto Superior Técnico, Lisboa, Portugal;

[2] Instituto de Investigação e Inovação em Saúde (i3S), Porto, Portugal;

[3] Institute of Molecular Pathology and Immunology of the University of Porto (IPATIMUP), Porto, Portugal;

[4] Department of Pathology and Oncology, Medical Faculty of the University of Porto, Porto, Portugal.

# These authors have equal contribution to the work
* Corresponding authors

SUPPLEMENTAL DATA

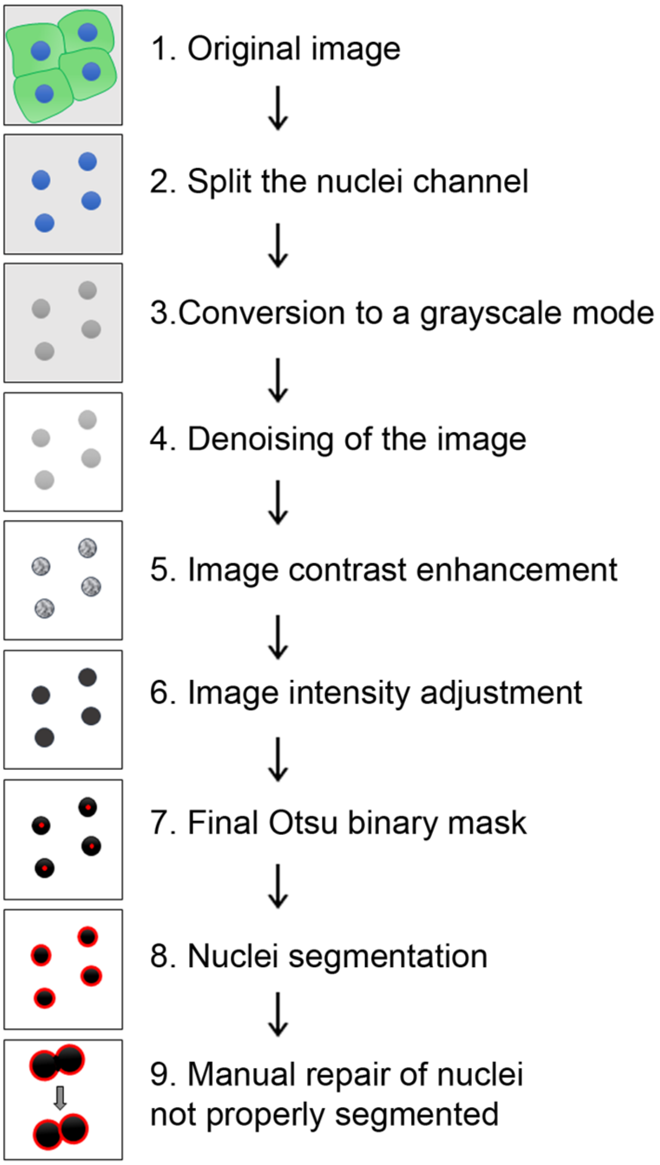

Supplementary Figure S1. Flowchart of image processing and nuclei segmentation procedures. The blue channel of an RGB original fluorescence image is extracted and converted to a gray-scale version. Upon denoising, the image is subjected to contrast and intensity adjustments. A final binary mask with the non-segmented nuclei is thus obtained. Finally, nuclei segmentation and manual fixation of nuclei not properly segmented is performed. For each step, a schematic representation is presented.
